# Supplementary figures and images for: C-Reactive protein as a prognostic indicator in hospitalized patients with COVID-19
Source: PLoS One. 2020 Nov 20;15(11):e0242400. doi: 10.1371/journal.pone.0242400 (PMC7679150; doi:10.1371/journal.pone.0242400)

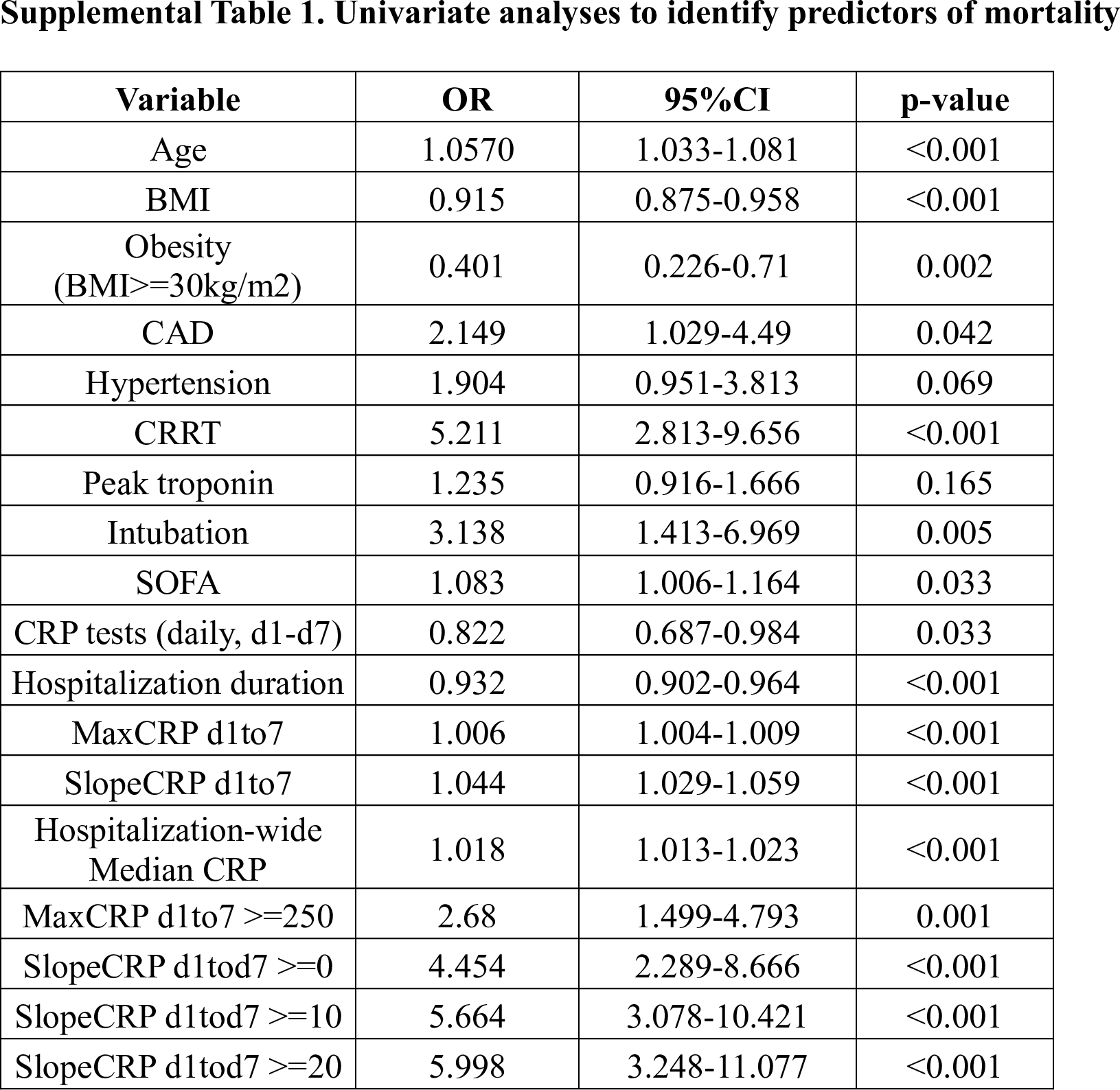

Supplement: S1 Table — (TIF) [file pone.0242400.s001.tif]
