# Supplementary material for: C-Reactive protein as a prognostic indicator in hospitalized patients with COVID-19
Source: PLoS One. 2020 Nov 20;15(11):e0242400. doi: 10.1371/journal.pone.0242400 (PMC7679150; doi:10.1371/journal.pone.0242400)
Supplement: Attachment — Submitted filename: Review Comments regarding manuscript entitled.docx [file pone.0242400.s002.docx]

**Review Comments regarding manuscript entitled: C-Reactive Protein as a Prognostic Indicator in Hospitalized Patients with COVID**

**Recommendation: Accepted we minor revision**

**I found the manuscript very interesting**

**Comments:**

- English editing
- Table 1 must be separated in to different tables: One for demographic characteristics and another for Hospitalization details
