# Supplementary material for: C-Reactive protein as a prognostic indicator in hospitalized patients with COVID-19
Source: PLoS One. 2020 Nov 20;15(11):e0242400. doi: 10.1371/journal.pone.0242400 (PMC7679150; doi:10.1371/journal.pone.0242400)
Supplement: Attachment — Submitted filename: Response to Reviewers.docx [file pone.0242400.s003.docx]

1. Please ensure that your manuscript meets PLOS ONE's style requirements, including those for file naming. The PLOS ONE style templates can be found at

https://journals.plos.org/plosone/s/file?id=wjVg/PLOSOne_formatting_sample_main_body.pdf and

<https://journals.plos.org/plosone/s/file?id=ba62/PLOSOne_formatting_sample_title_authors_affiliations.pdf>

Thank you! I have gone through the manuscript and to the best of my ability followed the PLOS ONE’s style requirements.

2. In your ethics statement in the Methods section and in the online submission form, please provide additional information about the data used in your retrospective study. Specifically, please ensure that you have discussed whether all data were fully anonymized before you accessed them and/or whether the IRB or ethics committee waived the requirement for informed consent. If patients provided informed written consent to have data from their medical records used in research, please include this information.

Thank you. The data were fully anonymized before we accessed them. Since this was a retrospective analysis, the IRB waived the requirement for informed consent.

3. Please provide the names of the six cohorted COVID ICUs across three Emory Healthcare acute care hospitals in Atlanta, GA.

1. 2 East ICU at Emory St. Joseph Hospital

2. Medical ICU at Emory University Hospital Midtown

3. Peachtree ICU at Emory University Hospital Midtown

4. 5G ICU at Emory University Hospital

5. 6G ICU at Emory University Hospital

6. 5E ICU at Emory University Hospital

4. Thank you for stating the following financial disclosure:

"No"

At this time, please address the following queries:

a. Please clarify the sources of funding (financial or material support) for your study. List the grants or organizations that supported your study, including funding received from your institution.
   a. This study was partly supported by internal funds (Department of Anesthesiology and Critical Care, Emory University Hospital)
   b. This study was also supported by the National Center for Advancing Translational Sciences of the National Institutes of Health under Award Number UL1TR002378.
      The content is solely the responsibility of the authors and does not necessarily represent the official views of the National Institutes of Health
b. State what role the funders took in the study. If the funders had no role in your study, please state: “The funders had no role in study design, data collection and analysis, decision to publish, or preparation of the manuscript.”
   a. The funders had no role in study design, data collection and analysis, decision to publish, or preparation of the manuscript.”
c. If any authors received a salary from any of your funders, please state which authors and which funders.
   a. None of the authors received a salary from the funder.
d. If you did not receive any funding for this study, please state: “The authors received no specific funding for this work.”
   a. Thank you very much. This is not applicable, and we have indicated our source of funding appropriately

Please include your amended statements within your cover letter; we will change the online submission form on your behalf.

5. One of the noted authors is a group or consortium [Emory COVID-19 Quality and Clinical Research Collaborative]. In addition to naming the author group, please list the individual authors and affiliations within this group in the acknowledgments section of your manuscript. Please also indicate clearly a lead author for this group along with a contact email address.

Emory COVID-19 Quality and Clinical Research Collaborative Members (in alphabetical order): Max W. Adelman, Scott Arno, Sara C. Auld, Theresa Barnes, William Bender, James M. Blum, Gaurav Budhrani, Stephanie Busby, Laurence Busse, Mark Caridi-Scheible, David Carpenter, Nikulkumar Chaudhari, Craig M. Coopersmith, Gordon Dale, Lisa Daniels, Johnathan A. Edwards, Jane Fazio, Babar Fiza, Eliana Gonzalez, Ria Gripaldo, Charles Grodzin, Robert Groff, Alfonso C. Hernandez-Romieu, Max Hockstein, Dan Hunt, Craig S. Jabaley, Jesse T. Jacob, Colleen Kraft, Greg S. Martin, Samer Melham, Nirja Mehta, Chelsea Modlin, David J. Murphy, Jung Park, Deepa Patel, Cindy Powell, Amit Prabhaker, Jeeyon Rim, Ramzy Rimawi, Chad Robichaux, Nicholas Scanlon, Milad Sharifpour, Bashar Staitieh, Michael Sterling, Jonathan Suarez, Colin Swenson, Nancy Thakkar, Alexander Truong, Hima Veeramachaneni, Alvaro Velasquez, Aimee Vester, Michael Waldmann, Max Weinmann, Thanushi Wynn, and Joel Zivot.

Dr. Sara Auld (sara.auld@emory.edu) is the lead author of the collaborative.

6. Please amend either the abstract on the online submission form (via Edit Submission) or the abstract in the manuscript so that they are identical.

 Thank you. I have corrected this and both abstracts are now identical.

7. We note you have included a table to which you do not refer in the text of your manuscript. Please ensure that you refer to Table 3 in your text; if accepted, production will need this reference to link the reader to the Table.

Thank you. We have included a sentence in the results section (line 187) as follows, referencing Table 2. ‘These thresholds for CRP measures are shown in Table 2. Table 3 was changed to Table 2 in order to keep the correct order of Tables in the body of the text.

8. Please include captions for your Supporting Information files at the end of your manuscript, and update any in-text citations to match accordingly. Please see our Supporting Information guidelines for more information: [http://journals.plos.org/plosone/s/supporting-information](about:blank).

1. In Table 1, please indicate the corresponding units for the variables (e.g., CRP, troponin, D-dimer, WBC). Please indicate the meaning of the acronyms for the clinical variables as well (e.g., DM, OSA, CAD, CRRT, CHF, CKD).

Thank you. These are reflected in the text/table now, as well as here.

CRP: mg/L

Troponin: ng/mL

D-Dimer: ng/mL

WBC: 10E3/mcL

DM: Diabetes Mellitus

CAD: Coronary Artery Disease

OSA: Obstructive Sleep Apnea

CRRT: Continuous Renal Replacement Therapy

CHF: Congestive Heart Failure

CKD: Chronic Kidney Disease

2. Table 2 shows two different models. Please indicate in the text what is the difference between them.

We have changed table 2 to table 3 and included a foot note to Table 3 to clarify the differences between Models 1 and 2 as follows:

Model 1: CRP slope and Maximum CRP levels during the first 7 days were used as continuous variables in the model.

Model 2: CRP slope≥0 and Maximum CRP level ≥ 250 were used as thresholds in the model.

3. Please indicate in Materials and Methods what was the methodology used to measure CRP levels and whether it was the same for all six hospitals.

All six hospitals use the same methodology to measure CRP. CRP reagent is used to measure CRP concentration by a turbidimetric method^1,2^ In the reaction, c-reactive protein combines with specific antibody to form insoluble antigen-antibody complexes. The Beckman Coulter SYNCHRON System(s) © (Beckman Coulter Life Sciences, Indianapolis, IN) automatically proportions the appropriate sample and reagent volumes into a cuvette. The ratio used is one part sample to 26 parts reagent. The system monitors the change in absorbance at 340 nanometers. This change in absorbance is proportional to the concentration of C-reactive protein in the sample and is used by the System to calculate and express C-reactive protein concentration based upon a single-point adjusted, pre-determined calibration curve

4. On lines 119-120, the word “one” is repeated. Did the authors mean “only one”?

Thank you, I have corrected this and eliminated the redundant “one” and changed it to “only one” in line 119.

References:

1. Boyden, A., Button, E., Germerog, D., "Precipitin Testing With Special Reference to the Measurement of Turbidity", J. Immunol., 57:211 (1947).

2. Hellsing, K., "The Effects of Different Polymers for Enhancement of the Antigen-Antibody Reaction as Measured with Nephelometry", Protides of the Biological Fluids, 23:579 (1973).
